# Seasonal variations in diet composition, diet breadth and dietary overlap between three commercially important fish species within a flood-pulse system: The Tonle Sap Lake (Cambodia)

**DOI:** 10.1371/journal.pone.0198848

**Published:** 2018-06-18

**Authors:** Kong Heng, Mathieu Chevalier, Sovan Lek, Pascal Laffaille

**Affiliations:** 1 EDB, Université de Toulouse, CNRS, ENFA, UPS, Toulouse, France; 2 EcoLab, Université de Toulouse, CNRS, INPT, UPS, Toulouse, France; 3 Department of Ecology, Swedish University of Agricultural Sciences, Uppsala, Sweden; Universitat Autonoma de Barcelona, SPAIN

## Abstract

Tropical lakes and their associated floodplain habitats are dynamic habitat mosaics strongly influenced by seasonal variations in hydrologic conditions. In flood-pulse systems, water level oscillations directly influence the connectivity to floodplain habitats for fish. Here, we aimed to investigate whether seasonal changes in the water level of a flood-pulse system (the Tonle Sap Lake, Cambodia) differentially affect diet breadth and dietary overlap of three common and commercially important fish species (*Anabas testudineus*, *Boesemania microplepis and Notopterus notopterus*) presenting important differences in their life-cycle (e.g. seasonal migration). For this purpose, the three fish species were sampled at four locations spread over the lake and their stomach contents extracted for analyses. Dietary differences were investigated across seasons regarding the diet composition and diet breadth of each species as well as the amount of dietary overlap between species. We found that the proportion of empty stomachs changed similarly across seasons for the three species, thus suggesting that ecological differences between species are not sufficient to outweigh the effect of seasonal variations in resource abundance. In contrast, changes in diet composition were species-specific and can be explained by ecological and behavioral differences between species. Diet breadth differed between species in all seasons, except during the wet season, and tended to be higher during the dry season when dietary overlap was the lowest. These variations likely result from changes in the diversity and amount of resources and may lead to habitat use shifts with potential implications for competitive interactions. In particular, increasing connectivity to floodplain habitats may reduce the competitive pressure during the wet season, while resource scarcity during the dry season may constrain individuals to diversify their diet to avoid competition. Overall, our results suggest a considerable plasticity in the feeding behavior of the three species as demonstrated by seasonal variation in both diet breadth and dietary overlap. Such variations can be explained by a number of factors and processes, including changes in resource availability or competitive interactions between individuals for resources, whose relative influence might vary depending on the magnitude and the timing of the flood-pulse driving the connectivity to floodplain habitats. Gaining knowledge on the seasonal evolution of fish’s diet is relevant for fisheries management and conservation and our result could be used to guide aquaculture development in Cambodia.

## Introduction

Seasonal change in hydrology is a prominent feature of freshwater ecosystems influencing populations, communities and ultimately ecosystem processes by modifying the connectivity to floodplain habitats [1]. Lateral connectivity influences many aspects of an organism’s life cycle as well as local community composition by regulating access to resources and habitats for spawning, growing and rearing [2,3]. However, the connectivity to floodplain habitats is currently undergoing strong human pressures through dam constructions and river channelization [4], with consequences on organisms and ecosystem processes [5]. For instance, by altering natural river flow and by dampening seasonal fluctuations and thus connectivity to floodplain habitats [5], dams have a strong influence on population and community composition [6]. Such pressures are even more problematic in flood-pulse systems where seasonal changes in hydrology are of critical importance to maintain the productivity and the biodiversity of these systems [7]. Indeed, several studies [8,9] have shown that the flood-pulse is the main factor structuring fish communities and that flood-pulse modification could impact fish populations [10] as well as among-species interactions [11].

Studying stomach contents provides useful information regarding trophic relationships between species [12] but also ecosystem functioning by evaluating resource use efficiency [13] and could be used to assist the development of management and conservation strategies in a multi-species framework [14]. Several species have been shown to shift their diet across seasons as a result of variations in the connectivity to floodplain habitats [15,16]. For instance, in the Amazon basin, a great number of fishes enter the flooded forest during the high water season to feed on fruits, seeds and other terrestrial resources [17,18]. Thus, changes in diet composition can reflect changes in the availability and the quantity of food resources. However, given that the same resource can be shared by numerous species and that each species can successively exploit different resources during the same year [19,20], changes in diet composition could also be attributed to intra- and interspecific competition [21–23]. For instance, during the season of low resource abundance, diet breadth could increase to reduce intraspecific competition [24], while dietary overlap among species may decrease to reduce interspecific competition [25,26]. Furthermore, if all resources do not change uniformly, individuals are expected to forage according to the optimal foraging theory, by seasonally exploiting the most profitable resources, leading to seasonal diet shift [27]. Another layer of complexity arises from the fact that the above mentioned patterns can vary depending on species characteristics. For instance, a large diet breadth is a frequently cited characteristic of invasive species, allowing them to thrive in a wide range of environments and to potentially exploit different resources depending on the season [28]. Therefore, studying how the diet of species varies by season within a multi-species framework may help unravel the processes involved within a given ecosystem and provide knowledge about the nature of interactions that exist between individuals and species.

The Tonle Sap Lake (TSL) is the largest natural lake in Southeast Asia and was designated as a UNESCO Biosphere Reserve in 1997. The TSL has a flood-pulse functioning and provides essential habitats for more than 242 fishes [29] and many endangered vertebrates [30–32]. The lateral connectivity between the main lake and its floodplain mainly depends on water coming from the Mekong River, representing more than 50% of the water balance. This connectivity is a key feature for many fishes and aquatic species, by providing access to habitats for rearing, spawning and growing [30,31], ultimately influencing community structure [29], system productivity [9] and population dynamics [33]. However, growing human pressures in the upper reaches of the Mekong River, through the establishment of large hydropower dams and reservoirs, large irrigation schemes, and rapid urban development, is putting water resources under stress and is strongly threatening the connectivity to floodplain habitats [4].

The aim of this study was to describe the seasonal variations of the diet of three common, abundant and commercially important fishes: *Anabas testudineus*, *Boesemania microplepis* and *Notopterus notopterus* [29]. We chose these species because they are widespread within the lake during the whole year and are known to occur in similar habitats [34], thus providing potential for interspecific competition. They nevertheless present important differences within their life-cycle, including contrasting seasonal migration patterns and tolerance to hypoxia [34–36]. To investigate seasonal variations in the diet composition and diet breadth of each species as well as the amount of dietary overlap between species, we analyzed the stomach contents of 623 specimens collected at four locations across four hydrological phases (dry, rising, wet and receding seasons) covering the dynamic of the TSL. Given seasonal changes in the water level and the lateral connectivity to floodplain habitats, our expectations were as follow. First, we hypothesized seasonal changes in the diet of the three species, regarding both the proportion of empty stomachs and the proportion of food items. In particular, we expected a higher proportion of empty stomachs during the dry season because of low resource abundance. We further hypothesized that the diet breadth of the three species would be larger during the dry season to reduce intraspecific competition for resources while it would be lower during the wet season because individuals would be able to forage on the most profitable resource due to an increase in both the availability and the diversity of food resources. Finally, we expected seasonal variations in dietary overlap and in particular a low dietary overlap during the dry season due to competitive exclusion for resources between species.

## Materials and methods

### Study species

*Anabas testudineus* is a demersal species, commonly found in sluggish, standing and stagnant waters with dense vegetation and mainly feed on shrimps and fish fry [37]. It belongs to the “*blackfish”* guild where fish move only locally from waterbodies to the surrounding floodplain during the wet season and return to the pools during the dry season. Those fishes are adapted to hypoxia and often present auxiliary respiratory organs that enable them to breathe atmospheric air or behaviors that give them access to the well-oxygenated surface films. They have a wide range of breeding behaviors that allows them to maintain eggs and newly hatched fry in relatively well-oxygenated localities.

*Boesemania microplepis* is a bentho-pelagic species, commonly found in flowing waters of large rivers and in the deep pools of the Mekong River even during the dry season [38]. It mainly feeds on crustaceans and small fishes [34]. This species is categorized as a “*whitefish”*, comprising large, strongly migratory fishes that move large distances within the river channels between feeding and breeding habitats. Fish within this guild may pass their whole life history in the main channel or may move into the floodplains to feed. They are generally intolerant to hypoxia, preferring migration as a means to escape the adverse conditions during the dry season. Whitefish are generally one-shot spawners, scattering numerous eggs.

*Notopterus notopterus* is a species found in a wide range of habitats including fresh waters, standing and sluggish waters, floodplains, canals, and ponds. It mainly feeds on insects, fishes, crustaceans and roots [34]. This species belongs to the “*grayfish”* guild which is intermediate between the floodplain-resident and the long-distance migrant’s guilds. Grayfish generally execute short migrations between the floodplain, where they reside during the wet season for breeding and feeding, to the main river channel, where they shelter in marginal vegetation or in the deeper pools of the channel over the dry season. These species are intolerant to hypoxia but present elaborate reproductive behaviors, which enable them to go into the floodplain for breeding during the dry season.

Given differences in the ecology and the behavior of the three species, specific patterns were expected. In particular, we hypothesized that *N*. *notopterus* would display low seasonal variations in its diet breadth because of its ability to migrate back and forth between different habitats, making it possible for individuals to forage on different resources and to maintain a broad diet during the whole year. *B*. *microplepis* tends to migrate to escape adverse conditions during the dry season and we therefore expected this species to present a low dietary overlap with respect to the two other species during this season. In contrast, we expected the diet of *A*. *testudineus* to change toward the most profitable resource (i.e. fish) during the dry season because of its ability to face adverse conditions and exclude other competitors.

### Study area

**Fig 1:**
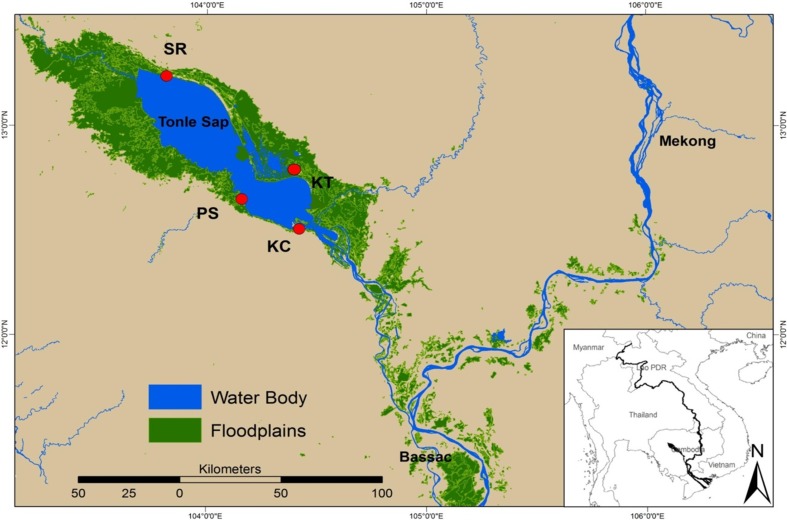
Map of the Tonle Sap Lake (TSL). Sampling locations are indicated by red dots. SR = Siem Reap, KT = Kampong Thom, PS = Pursat and KC = Kampong Chhnang.

Located in the central part of Cambodia, the TSL is the largest natural lake in Southeast Asia (Fig 1). During the dry season it covers an area of approximately 0.25 million hectares whereas during the peak flood in the wet season the area covered by the lake has been estimated between 1.0 to 1.3 million hectares. The TSL is connected to the Mekong River in its southern part by a 120 km long river, the Tonle Sap River (TSR) which serves as an inlet and outlet for water fluxes. The TSL’s hydrological cycle can be divided into four phases [29]. The first phase (rising season) takes place from July to early September and is characterized by a strong water feed coming from the upper Mekong through the TSR and during which the water level strongly increases. The second phase (the wet season) lasts from the end of September to the end of October, and corresponds to a phase where about 1.25 million hectares of forest, shrublands, grasslands and agricultural lands are submerged. At this period the water level may attain up to 15 meters. The third phase (the receding season) occurs from the end of October to February, and is characterized by the reversal of the TSR flowing from the TSL to the China Sea in the south, resulting in a decrease of the water level of the lake. Finally, the fourth phase (the dry season) lasts from April to May, and corresponds to the period where the water level is the lowest (i.e. 1 to 2 m).

### Data collection and dietary analysis

Data collection was carried out in collaboration with the Inland Fisheries Research and Development Institute (IFReDI) who issued the permission for collection and were collected in strict accordance with the Cambodian Fisheries Law on small-scale fishing. None of the studied species are classified as either endangered or protected according to the IUCN red list.

To match with the four natural phases of the TSL’s hydrological cycle, fish were sampled every three months from the beginning of June 2014 to the end of May 2015 by local fishermen using gillnets with varying mesh sizes (2 to 6.5 cm), heights (1.5 to 2 m) and lengths (250 to 300 m), to capture individuals with varying sizes. Gillnets were deployed during the night and were let in the water for eight to 10 hours. The habitat where the nets were set varied depending on the water level. During the wet and the receding seasons, nets were set within floodplain habitats (i.e. in the middle of shrubs, grasslands and trees) whereas during the dry and the rising seasons, nets were set in open water habitats as floodplain habitats are not accessible at this period of the year. To account for spatial variation in resource distribution within the TSL and low local abundance in some cases, the specimens were collected at four locations (Fig 1): Kompong Thom (KT), Siem Reap (SR), Pursat (PS) and Kompong Chhnang (KC). The three species were always found within the same nets, confirming that they share similar habitats and that they potentially compete for resources. The number of individuals associated to each species varied depending on the location and the season (Table 1). To avoid the breakdown of food items, specimens were immediately preserved in a cool container, and stomach contents were extracted at the laboratory and preserved in 97% ethanol. Thus, the death of fish specimens was induced by thermal shock, as recommended [39,40]. The proportion of empty stomachs was scored for each species and seasons. From the remaining stomachs, food items were identified to the lowest feasible taxonomic level following [41] and [42] using optical and stereoscopic microscopes (Olympus: SZX7-Model SZX2-ILLK). For each food item, we calculated its frequency of occurrence (FOC) as the number of times a particular item occurred within a sample relative to the total number of items (empty stomach excluded). Among the four sampling campaigns, a total of 623 specimens were collected and analyzed for stomach contents (236 for *A*. *testudineus*, 179 for *B*. *microplepis* and 208 for *N*. *notopterus*). In total, 30 different prey types were found within the non-empty stomachs of the three species.

**Table 1:** Total number of individuals of the three species collected (i) at each location across the four seasons and (ii) during each season across the four locations.

Species	*Locations*				*Seasons*			
	KT	SR	KC	PS	Receding	Wet	Rising	Dry
*Anabas testudineus*	55	83	50	48	80	56	35	65
*Boesemania microlepis*	30	51	59	39	65	31	6	77
*Notopterus notopterus*	35	69	48	56	78	34	23	79

Abbreviations for locations: KT = Kompong Thom, SR = Siem Reap, PS = Pursat and KC = Kompong Chhnang.

### Statistical analysis

Because the proportion of empty stomachs can provide information on resource availability and the ability of species to forage during periods of shortage [12], we tested for seasonal variations in the proportion of empty stomachs using a chi-square test.

For non-empty stomachs, we used an analysis of similarity (ANOSIM) to test for significant differences in the diet of species between the wet (from July to October) and the dry seasons (from December to May) followed by an analysis of similarity percentages (SIMPER) to assess the contribution of food items to the observed variations [43]. We focused on differences between the wet and the dry season because considering the four seasons would have implied to perform multiple tests (i.e. six for each species), necessitating p-values adjustments and thus a decrease in statistical power. Both analyses were based on a dissimilarity matrix built with Bray-Curtis distances to account for the large proportion of zeros in the community matrix. The ANOSIM statistic vary between zero and one and compares the mean of ranked dissimilarities between groups to the mean of ranked dissimilarities within groups. A value close to one suggests dissimilarity between groups, while a value close to zero suggests an even distribution of high and low ranks within and between groups. The SIMPER analysis is based on the decomposition of the Bray-Curtis dissimilarity matrix and aim to assess the contribution of food items (or species) to the observed dissimilarities. For this analysis, we grouped food items into six broader categories (fish, insects, crustaceans, plants, mollusks and micro-fauna) following [22,44]. The importance of each category within the diet was established as its average FOC divided by the sum of the average FOC of all categories and was calculated by species and seasons.

To explore interspecific differences in diet composition across the four seasons, we used non-metric multidimensional scaling (NMDS [45]). This indirect gradient analysis approach uses rank order values to visualize and interpret differences between species, ultimately representing pairwise differences between samples in a low-dimensional space. The similarity between sampling units was calculated using Bray-Curtis distances. Beyond this graphical approach, we computed different statistics to evaluate and test how interspecific differences in diet varied across seasons. First, we used a multivariate homogeneity of group dispersions [46] with 1000 permutations to (i) measure the diet breadth of each species in each season and (ii) test for interspecific differences in each season. In this analysis, diet breadth is estimated as the average distance of prey items to the group centroid within the multivariate space described by the NMDS with larger values indicative of a broader diet. The values by themselves have no ecological meaning but can be compared to determine to which extent diet breadth is changing between species and season. Second, we computed Pianka’s symmetric index [47] to measure niche overlap in diet composition between each pair of species in each season. Here, a value close to zero indicates no overlap whereas a value close to one indicates a strong overlap. To test the statistical significance of dietary overlap between each pair of species in each season, we conducted a bootstrap procedure by randomly sampling the rows of the community matrix. This procedure was repeated 1000 times and generated a distribution of Pianka’s index for each pair of species under the null hypothesis that there is no variation in dietary overlap across seasons. For a given pair of species, we assessed the statistical significance of dietary overlap in each season by comparing the observed value to the 95% confidence intervals of the corresponded distribution. All analyses were performed in R [48] using the package vegan [49].

## Results

**Fig 2:**
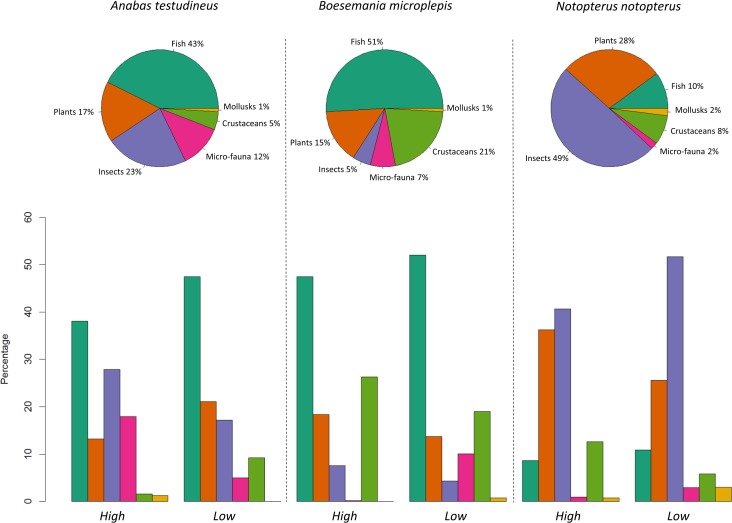
Description of the diet of the three studied species and how they vary across seasons. For the sake of graphical clarity and because some items had a very low occurrence, items were grouped into six broader categories (mollusks, crustaceans, fish, plants, insects and micro-fauna). The pie charts represent the relative proportion of food items, all seasons confounded, and thus provide an overview of the main diet of the species. The barplots represent the seasonal variations in the proportion of food items within non-empty stomachs (colored barplots; on the left) and the seasonal variations in the proportion of empty stomachs (greyed barplots; on the right).

The average size of captured individuals was 12.7 ± 2.1 cm for *A*. *testudineus*, 23.9 ± 13.9 cm for *B*. *microplepis* and 20.4 ± 2.4 cm for *N*. *notopterus*. The proportion of empty stomachs was 14.8% for *A*. *testudineus*, 12.3% for *B*. *microplepis* and 16.8% for *N*. *notopterus*. For all species, this proportion greatly varied across seasons and was maximal during the receding season and minimal during the dry season (Fig 2). Seasonal variation in the proportion of empty stomachs was independent of species identity (χ^2^ = 0.14, df = 6: P = 0.99). The diet of *A*. *testudineus* was mainly composed of fish (43%), insects (23%) and plants (17%) whereas *N*. *notopterus* mostly fed upon insects (49%), plants (28%) and fish (10%) (Fig 2). The diet of *B*. *microplepis* was mainly composed of fish (51%), crustaceans (21%), and plants (15%). The similarity analysis (ANOSIM) revealed a significant change in the diet composition of *A*. *testudineus* (*p*<0.01, r = 0.16) and *N*. *notopterus* (*p*<0.01, r = 0.13) between the wet and the dry season, whereas no significant difference was found for *B*. *microlepis* (*p* = 0.42, r = 0.004). The SIMPER analysis revealed that only a few food items significantly explained the seasonal differences found for *A*. *testudineus* and *N*. *notopterus*, with a particularly strong contribution of fish prey for the former and of insects for the latter (Table 2).

**Table 2:** Average percent contribution of food items to the dissimilarity of diet composition between the wet and the dry seasons for the three studied species.

Food items	*A*. *TESTUDINEUS*	*B*. *MICROLEPIS*	*N*. *NOTOPTERUS*
Fish	0.28 (0.25)	0.28 (0.22)	0.07 (0.11)
Plants	0.11 (0.13)	0.09 (0.09)	0.12 (0.11)
Insects	0.17 (0.19)	0.05 (0.1)	0.20 (0.15)
Micro-fauna	0.13 (0.14)	0.05 (0.06)	0.02 (0.05)
Crustaceans	0.04 (0.13)	0.17 (0.19)	0.07 (0.13)
Mollusks	0.01 (0.03)	0.01 (0.01)	0.02 (0.04)

Only for *A*. *testudineus* and *N*. *notopterus* is there a significant contribution of food items to the seasonal dissimilarity of diet composition. Numbers within brackets are standard deviations.

**Fig 3:**
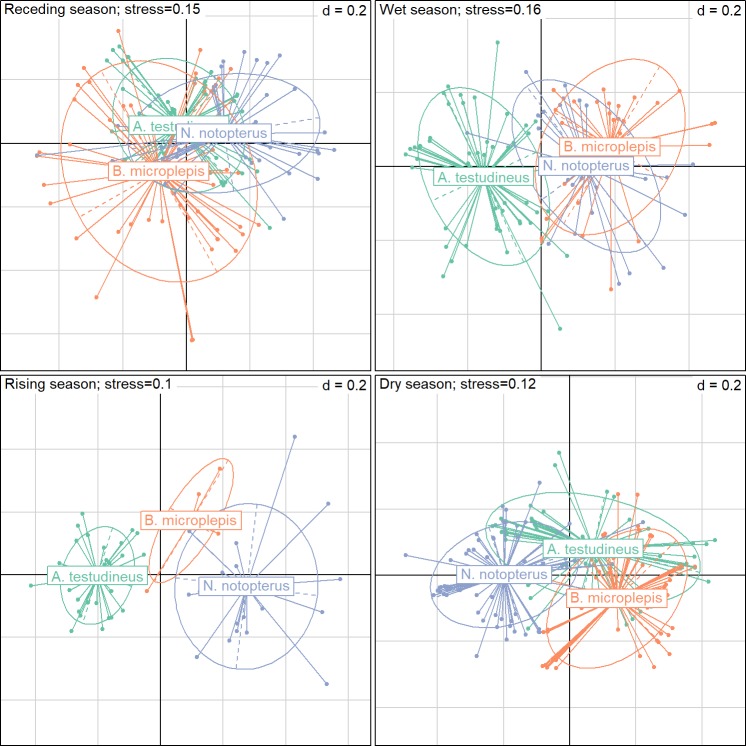
NMDS biplots displaying the diet of the three species across the four seasons. The size of the ellipse represent species diet breadth whereas overlap between ellipses relates to dietary overlap between species.

The NMDS highlighted strong seasonal variations in both diet breadth and dietary overlap between the three species (Fig 3). Diet breadth significantly differed between the three species in all seasons (*p*<0.01), except the wet season (*p* = 0.11; Table 3). It tended to be highest during the dry (from 0.48 to 0.54) and the receding seasons (from 0.45 to 0.53), and to be lowest during the two remaining phases (from 0.34 to 0.51) of the hydrologic cycle (Table 3). From a species perspective, diet breadth was quite stable across seasons for *N*. *notopterus* (from 0.46 to 0.51) but much more variable for the two other species, with variations from 0.38 to 0.54 for *A*. *testudineus* and from 0.34 to 0.53 for *B*. *microlepis* (Table 3).

**Table 3:** Diet breadth estimate for the three studies species across the four seasons. *p*-values were obtained using 1000 permutations on the community matrix. Values inferior to 0.05 point to a significant difference in the diet breadth of the three species.

Hydrological phase	*A*. *TESTUDINEUS*	*B*. *MICROLEPIS*	*N*. *NOTOPTERUS*	P-VALUE
Receding season	0.45	0.53	0.47	0.002
Wet season	0.48	0.42	0.46	0.115
Rising season	0.38	0.34	0.51	0.004
Dry season	0.54	0.48	0.49	0.005

**Fig 4:**
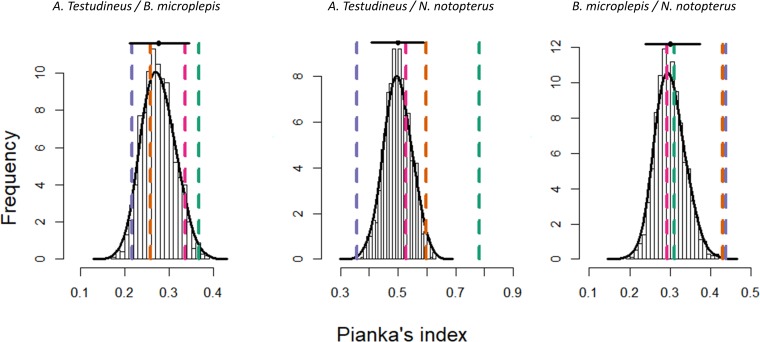
Results from the bootstrap procedure conducted on Pianka’s symmetric index, measuring the degree of niche overlap between the diets of two species. Histograms present the generated distribution of Pianka’s index between two species under the null hypothesis that there is no seasonal variation in dietary overlap. At the top of each histogram, the black horizontal bar represents the 95% confidence interval of the distribution while the black dot represents the average of the distribution. Colored vertical dashed lines point to the observed measure of niche overlap between two species in each season: receding season = green; wet season = orange; rising season = purple; dry season = pink. Any vertical line not overlapping the horizontal black line indicate a significant difference in the niche overlap for the corresponding season.

The bootstrap procedure performed on Pianka’s index suggest that dietary overlap was different between the three species. This was revealed by differences in the null distributions, whose averages vary from 0.27 [95% CI = 0.21–0.34] between *A*. *testudineus* and *B*. *microlepis* to 0.5 [95% CI = 0.41–0.58] between *A*. *testudineus* and *N*. *notopterus*, while it was 0.3 [95% CI = 0.23–0.37] between *B*. *microlepis* and *N*. *notopterus* (Fig 4). Confidence intervals further suggest that the dietary overlap measured between *A*. *testudineus* and *N*. *notopterus* is significantly higher than the one measured between the two other pair of species (non-overlapping confidence intervals). We found evidence for seasonal variations in dietary overlap with minimum values observed during the rising season and maximum values observed during the receding season. In particular, dietary overlap between *A*. *testudineus* and *N*. *notopterus* was significantly higher during both the receding and the wet seasons, whereas it was significantly lower during the rising season (all *p*<0.05; Fig 4). Likewise, dietary overlap between *B*. *microlepis* and *N*. *notopterus* was significantly higher during both the rising and the wet seasons (both *p*<0.001; Fig 4). Dietary overlap between *A*. *testudineus* and *B*. *microlepis* was more stable across seasons, but was significantly higher than expected in the receding season (*p*<0.01; Fig 4).

## Discussion

In this study, we explored seasonal variations in the diet of three common, abundant and commercially important fish species presenting important differences within their life-cycle but sharing similar habitats to gain knowledge about resource use and the potential for resource competition. Although we found large seasonal variations in the proportion of empty stomachs, we found no differences between species in this regard. In contrast, we found strong seasonal variations in the diet breadth and dietary overlap of the three species. Diet breadth was the largest during the dry and the receding seasons whereas dietary overlap between species was the lowest during the dry season. We also found evidence for seasonal variations in the diet of *A*. *testudineus* and *N*. *notopterus* with a differential contribution of food items, while no significant change was found for *B*. *microlepis*.

A similar seasonal variation in the proportion of empty stomachs suggests that ecological differences between species (e.g. reproductive behavior, foraging abilities) are probably not strong enough to outweigh the effect of seasonal variations in resource abundance. For the three species, the proportion of empty stomachs was the lowest during the dry season (i.e. when floodplain habitats are unachievable) but the highest during the receding season, which contrast to our expectation. One possible explanation would be that the high abundance and diversity of fish during the receding season, combined with the reversal of the river flow, that sweeps out resources from the system, would increase competition for resources, thereby decreasing the *per capita* consumption rate [50]. In contrast, the low abundance and diversity of fish during the dry season might contribute to increase the species *per capita* consumption rate, although the amount of resources is lower relative to the receding season. Another, non-exclusive explanation would be that during the receding season, individuals rely on reserve energy stored during the wet season, while during the dry season, individuals have to maximize their energy intake to face an increase in metabolic demand caused by adverse conditions such as oxygen depletion [12].

In contrast to the proportion of empty stomachs, we found differences in the seasonal variation of the stomach contents of the three species. Such variations likely result from temporary connections to floodplain habitats caused by expansion-contraction cycles of the TSL’s flooded area, providing access to new resources [51,52]. For instance, the increase in the proportion of crustaceans and insects in the diet of *B*. *microlepis* between the dry and the rising seasons can be explained by the progressive connection to floodplain habitats making it possible for individuals to forage in previously unflooded areas where those items are rather abundant. Similar changes were observed within other tropical systems where seasonality was shown to influence fish diet through an increase in the frequency of particular food items such as terrestrial insects and amphipods during the rainy season [53]. In line with our expectation, we found that fish prey contributed strongly to seasonal variations in the diet of *A*. *testudineus* with a substantial increase (i.e. around 10%) during the dry season. This suggest that the ability for this species to face adverse conditions (i.e. low levels of oxygen) makes it possible for individuals to exclude other competitors and to forage on the most profitable resource independently of the connection to floodplain habitats [54].

In line with our expectation and with previous findings [55], we found that the diet breadth of the three species tended to be larger during the dry (and the receding) season. This result could be explained by an increase in both intra and interspecific competition, constraining individuals to adopt an opportunistic strategy and diversify their diet to reduce competition [24]. The diet breadth of the three species differed in all seasons, except the wet season. This lack of difference was due to an increase in the diet breadth of *A*. *testudineus* and *B*. *microlepis* combined with a decrease in the diet breadth of *N*. *notopterus* between the rising and the wet seasons, leading to a similar diet breadth between the three species (from 0.42 to 0.48). The increase observed for the first two species can be explained by an increase in the diversity and amount of resources combined with a release of the competitive pressure, making it possible for those species to diversify their diet. In contrast, the decrease observed for the last species can be explained by its ability to forage on a large array of resources during periods of shortage (habitat generalist) while during periods of resource abundance individuals can focus on the most profitable resource. As expected, we found large seasonal variations in the diet breadth of *A*. *testudineus* and *B*. *microlepis* but low variations in the diet breadth of *N*. *notopterus*. The large variations observed for *A*. *testudineus* and *B*. *microlepis* are in accordance with their ecological status (invasive for the former and long-distant migrant for the latter), making it possible for these species to adapt their diet as the amount and the diversity of resources changes over time or space. In contrast, the large and stable diet breadth observed in *N*. *notopterus* could be attributed to its high mobility between diverse habitats that potentially contain different resources.

Regarding temporal changes in the availability, quantity and quality of food resources, species are expected to adjust their foraging behavior in order to maximize their energy intake and minimize dietary overlap [52]. In this study, we found an overall high dietary overlap between species during the receding season (e.g. between *A*. *testudineus* and *N*. *notopterus)* and a lower dietary overlap during the rising season, particularly when *B*. *microlepis* was involved in the comparison, in accordance with our expectation. The pattern observed during the receding season can be explained by the progressive loss of resources, as the lake retracts, whereas the one observed during the rising season can be explained by the progressive connection to floodplain habitats providing opportunities for species to forage on different resources. Contrasted results have however been reported in the literature. For instance, some studies have found that dietary overlap tends to be the lowest during the dry season because fish tend to concentrate in small, well-oxygenated, areas [26,56], whereas others [17,21,25] found the opposite (i.e. high dietary overlap during the dry season). In flood-pulse systems, fish assemblages and diet composition have been shown to differ with respect to habitat heterogeneity, hydrological conditions and connectivity between adjacent systems [57] while the intensity and the duration of the flood pulse have also been shown to strongly influence dietary overlap between species [11]. The absence of general pattern suggests that seasonal changes in trophic dynamics are not fully explained by feeding strategies, that aim to reduce competition between species and individuals, but that feeding opportunism may be an important underlying factor [21]. The discrepancies existing between previous studies and this one reflect the fact that different patterns can occur depending on the characteristics of the system, including its productivity, the nature of resources (e.g. autochtonous *vs*. allochtonous) and the characteristics of the species (e.g. generalist *vs*. specialist).

## Concluding remarks

This study is based on stomach contents analyses, for which a number of issues have been identified [58]. Indeed, stomach contents only reflect a “snapshot” of an individual's recent diet and can be highly variable among individuals [59]. Furthermore, stomach contents are usually biased toward recent dietary items and prey that does not readily digest, such as zooplankton, and can underestimate the amount of other preys, such as fish [60]. Differential digestion rates between species may also have an influence on the estimation of dietary overlap [58]. Likewise, since larger individuals tend to have larger stomachs, variations in diet composition mainly reflects variations in adult diets while among-species differences in diet breadth can be influenced by differences in species’ sizes. Nonetheless, these issues should have a minimal influence on our results because (i) we have sampled individuals of various sizes in different locations, thus ensuring that we have a global overview of the diet of the different species and (ii) our analyses are based on the frequency of occurrence and thus are not influenced by the abundance of food items relative to other individuals or species. An alternative to stomach content analyses is to use stable isotopes of Carbon and Nitrogen to quantify the dietary composition of individuals [61]. However, limitations have also been raised regarding this approach [62] and it seems that the best way to proceed is actually to use both approaches as complementary insights can be gained relative to when they are used separately [60,61].

The strong seasonal variations highlighted here with respect to diet composition, diet breadth and dietary overlap can be explained by a variety of mechanisms including changes in resource abundance and diversity, changes in the strength of competitive interactions between individuals and species as well as changes in ontogeny [63]. Teasing apart the relative influence of these factors is impossible with our data. The collection of additional data across seasons, for various organisms belonging to different trophic compartments (e.g. phytoplankton, zooplankton, invertebrates) and age classes (reflecting variable development stage) and in multiple locations spread over the lake (especially floodplain habitats) would help gain knowledge about (i) the nature of interactions between species and individuals and (ii) the extent to which they can shift their diet as the connectivity to floodplain habitats is changing. Such a study would not only improve our understanding of the mechanisms promoting biodiversity within flood-pulse systems but might also help guide management strategies within the Tonle Sap.

Overall, our results suggest that the flood-pulse may play a role in mediating the competitive interactions between the three species by making it possible for species to shift their diet as the availability of resources changes over time. This may ultimately promote biodiversity by providing opportunities for species to avoid competition and live in harmony with other species displaying similar dietary requirements during some periods of the year. This harmony is however threatened by accelerating water infrastructure development (hydropower, irrigation, flood control, and water supply) and climate change, bringing considerable modifications to the flood pulse of the Tonle Sap Lake in the foreseeable future.
